# Unstart Coupling Mechanism Analysis of Multiple-Modules Hypersonic Inlet

**DOI:** 10.1155/2013/254376

**Published:** 2013-11-14

**Authors:** Jichao Hu, Juntao Chang, Lei Wang, Shibin Cao, Wen Bao

**Affiliations:** ^1^School of Energy Science and Engineering, Harbin Institute of Technology, Harbin 150001, China; ^2^Academy of Fundamental and Interdisciplinary Sciences, Harbin Institute of Technology, Harbin 150001, China

## Abstract

The combination of multiplemodules in parallel manner is an important way to achieve the much higher thrust of scramjet engine. For the multiple-modules scramjet engine, when inlet unstarted oscillatory flow appears in a single-module engine due to high backpressure, how to interact with each module by massflow spillage, and whether inlet unstart occurs in other modules are important issues. The unstarted flowfield and coupling characteristic for a three-module hypersonic inlet caused by center module II and side module III were, conducted respectively. The results indicate that the other two hypersonic inlets are forced into unstarted flow when unstarted phenomenon appears on a single-module hypersonic inlet due to high backpressure, and the reversed flow in the isolator dominates the formation, expansion, shrinkage, and disappearance of the vortexes, and thus, it is the major factor of unstart coupling of multiple-modules hypersonic inlet. The coupling effect among multiple modules makes hypersonic inlet be more likely unstarted.

## 1. Introduction

The performance of a ramjet/scramjet powered hypersonic vehicle is determined by its inlet efficiency. Specifically, the inlet shock wave system influences the compression efficiency, mass capturing, and combustion stability. Unstart phenomenon is one of the most important issues of hypersonic inlets. For hypersonic air-breathing engines, inlet unstart causes a large drop both engine thrust and specific impulse, and thus, it may cause catastrophic damage during hypersonic flight [1]. The hypersonic inlet unstart can be generated by either the design factors (such as large internal area-contract ratios and serious shock/boundary-layer interactions) or the operation conditions (such as low flight Mach number, large angle of attack, and high backpressure). It is a challenge to ensure that a hypersonic inlet is always started. In the existing flight tests, including CIAM/NASA in 1999 [2], Hypersonic Collaborative Australia/United States Experiment in 2008 [3], X-51A in 2011 [4], inlet unstart occurred due to various reasons. Therefore, inlet unstart should be studied deeply and seriously.

There have been many papers devoted to hypersonic inlet unstart, while most of literature studies treat it as a steady state flow phenomenon [5–9] Yu et al. [5–7] discussed the feature selection and; pattern classification of hypersonic inlet start/unstart based on numerical and experimental data, Chang et al. [8] studied the effect of boundary layer bleeding on unstart/restart characteristics of hypersonic inlet. Only a few papers [10–13] considered it as an unsteady flow phenomenon using some transient testing methods. Tan et al. [13] firstly investigated the oscillatory flow of hypersonic inlets caused by the downstream massflow choking, and the results suggest that hypersonic inlets usually suffer from violent shock wave system oscillations, prominent pressure fluctuations, and abrupt performance reductions when unstarted phenomenon appears. Furthermore, the surface pressure shows a significant periodic pressure oscillation during the process of hypersonic inlet buzz, which causes unsteady aerodynamic loads. And they are highly detrimental to the structural safety of scramjets and the flight control of vehicles.

Multiple-modules in parallel manner are an important way to achieve the much higher thrust for scramjet engines. The modular nature facilitates engine scaling. An engine can be easily scaled by adding more flowpaths without requiring fueling redistribution because each flowpath is identical. In addition, scaling in this manner does not change the boundary-layer characteristics relative to the flowpath geometry. Aerojet conducted this multiple-modules strutjet engine [14] in this parallel manner. The advantage of using this approach is that each module is independent and the geometry and flowfield of scramjet engine are not needed to be redesigned, and it is beneficial to the rapid prototyping of scramjet engine.

For multiple-modules engines, when inlet unstart appears in a single-module engine due to high backpressure, how to interact with each module by the massflow spillage, and whether inlet unstart phenomenon occurs in other modules are important issues, and they are also the investigation emphasis of this paper. To discuss this problem, inlet model and numerical method are given in Section 2.  Section 3 presents some typical simulation results and analysis.  Section 4 presents some conclusions.

## 2. Inlet Model and Numerical Method

### 2.1. Inlet Model

**Figure 1:**
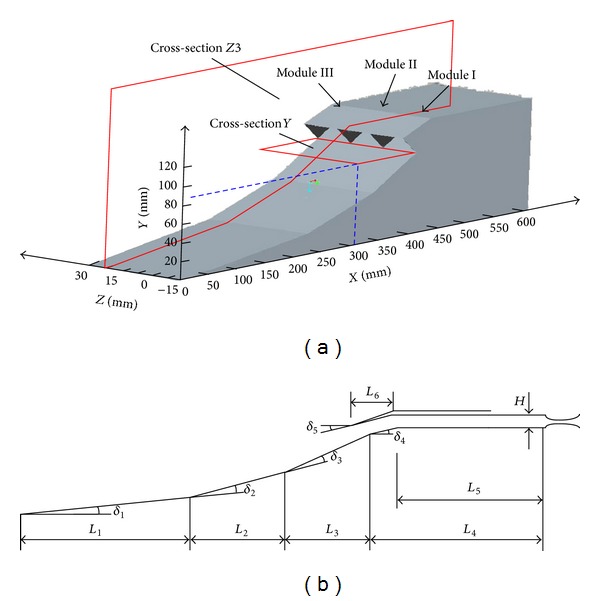
The 3D view of multiple-modules hypersonic inlet and geometric parameters.

The 3D view of a multiple-modules hypersonic inlet is presented in Figure 1. The duct of the multiple-modules hypersonic inlet is separated into three modules, module I, module II, and module III.

The multiple-modules hypersonic inlet is designed as a mixed-compression type, including three external shocks and two internal shocks. The main geometric parameters of the hypersonic inlet are referred to Figure 1 and Table 1, and the length unit is m and the angle unit is degree. The deflection angle of the three external ramps is 6°, 8.3°,and 9.8° respectively, and the deflection angle of the internal ramp and cowl tip is 10° and 14.1°, respectively. These compression ramps can guarantee the formation of a set of similar strength shock. The external contraction ratio and internal one are 5.47 and 1.29, so the total one is 7.05.

To show and analyze the flowfield structure conveniently in Section 3, some typical cross-sections are defined. Cross-section *Z*1, cross section *Z*2, and cross section *Z*3 parallel to *XY* plane, and their values of *Z*-axis are −7.5 mm, 7.5 mm, and 22.5 mm, respectively. Cross section *Y* parallels *XZ* plane, just located at the inlet entrance. These typical cross sections will be used to analyze the unstart coupling mechanism of the multiple-modules hypersonic inlet.

### 2.2. Numerical Method

The computation is performed using the finite-volume technique with upwind discretization to solve the three-dimensional compressible Reynolds-Averaged Navier-Stokes equations. The fluid was modeled as a single-specie, thermally perfect air. The piecewise-polynomial method was selected to compute specific heat while viscosity was solved using Sutherland's formula. The space discretization is performed by a cell-centered formulation. The advection upstream splitting method (AUSM) flux vector splitting is applied for the approximation of the convective flux functions. Higher-order accuracy for the upwind discretization and consistency with the central differences used for the diffusive term is achieved by the monotonic upstream scheme for conservation laws extrapolations, and the total variation diminishing property of the scheme is ensured by the Van Leer flux limiter. For transient simulations, the governing equations must be discretized in both space and time. Temporal discretization involves the integration of every term in the differential equations over a time step. Time integration is performed by an implicit five stage Runge-Kutta time-stepping scheme in the computation, and the advantage of the fully implicit scheme is that it is unconditionally stable with respect to time step size.

The computations have been performed using a structured grid. An excellent quality all-quad 3D mesh, with 2000 k control volumes, is generated for the simulation and ensuring the maximum *y* ^+^ was below 10 required for using with Shear-Stress Transport (SST) turbulence mode which is found to be the feasible turbulence model option for the simulation of vortices embedded in a boundary layer. For the steady simulation, the mass conservation is checked by measuring the mass-flow rate error. In general, 0.5% error is a good indicator for convergence.

The boundary condition of the supersonic inflow is far-pressure field, and the freestream conditions can be defined by specifying the boundary conditions. The boundary condition of the exit of the isolator is the pressure outlet, and the backpressure can be defined by changing the throttling area shown in Figure 1. At solid walls, the no-slip boundary condition is enforced by setting the velocity components to zero.

### 2.3. Numerical Accuracy Analysis

**Figure 2:**
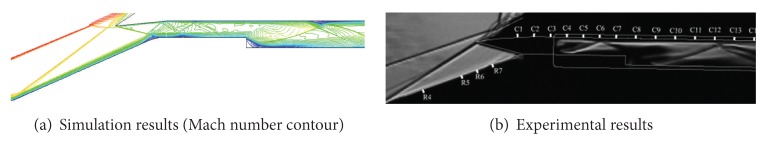
Comparison between Mach number contour and Schlieren pictures of a started inlet.

**Figure 3:**
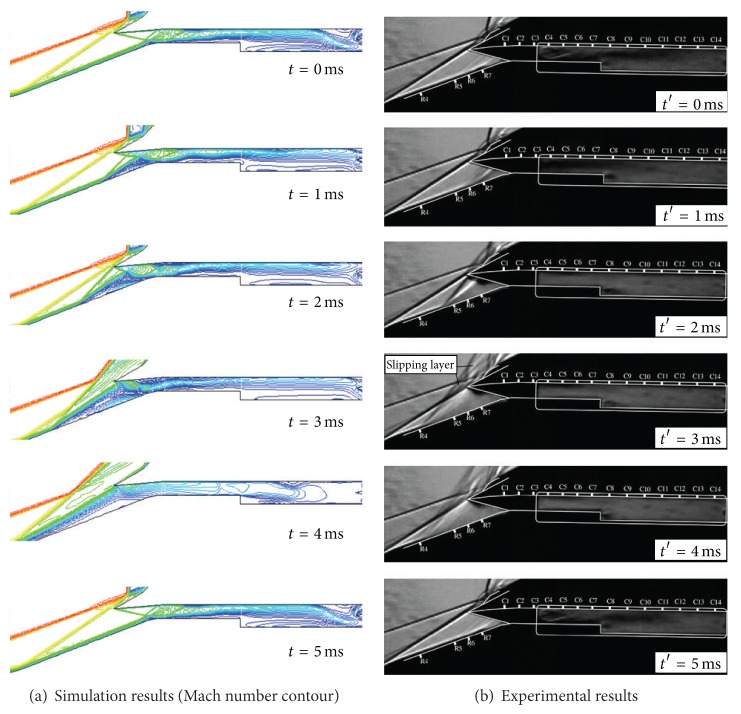
Comparison between Mach number contour and Schlieren pictures in a big buzz cycle.

Firstly, comparison of a Schlieren picture [13] without throttling and corresponding Mach number contour lines of the computation is shown in Figure 2, and it reveals an overall good agreement. The shock wave pattern, the separation, and the approximate boundary-layer thickness of the Schlieren picture are also present in the simulation results. Secondly, comparison of a Schlieren picture [13] with higher throttling ratio and corresponding Mach number contour lines of the computation is shown in Figure 3. At a higher throttling ratio, the hypersonic inlet buzz appears. It can be seen that during a big buzz cycle the inlet flow patterns exhibit a series of striking changes in contrast with a little buzz. The external compression shock system is first destroyed and then reestablished, the entrance separation bubble is first disgorged and then swallowed, and the shock train in the isolator is first developed and then dispelled.

As discussed above, the comparison between the simulation and experimental results shows that the simulation results accord with the physical conception of the aerodynamics and can reveal the primary characteristics of started and unstarted hypersonic inlet.

## 3. Results and Discussion

Whether inlet unstart phenomenon occurs or not depends on the mass flow rate balance between inlet entrance and combustor exit. If Mach number of the freestream is lower or the combustor backpressure is higher, inlet unstart would appear. For the multiple-modules hypersonic inlet, on the one hand, the difference of forebody shock compression of each module hypersonic inlet and the variation of angle of attack/sideslip angle all lead to the nonuniform of the flowfield at the entrance of isolators; on the other hand, the combustion oscillation or deflagration all result in that the pressure at the exit of isolators shows uneven. In contrast with a single-module engine, the factors discussed above increase the matching difficulty between the inlet and combustor characteristics for multiple-modules scramjet engines. The emphasis of this paper is to investigate the unstart coupling mechanism of multiple-modules hypersonic inlet due to the backpressure of a single-module hypersonic inlet, especially on how to interact with each module by the massflow spillage. 

### 3.1. Unstarted Flowfield Structure Caused by Module II Throttling

**Figure 4:**
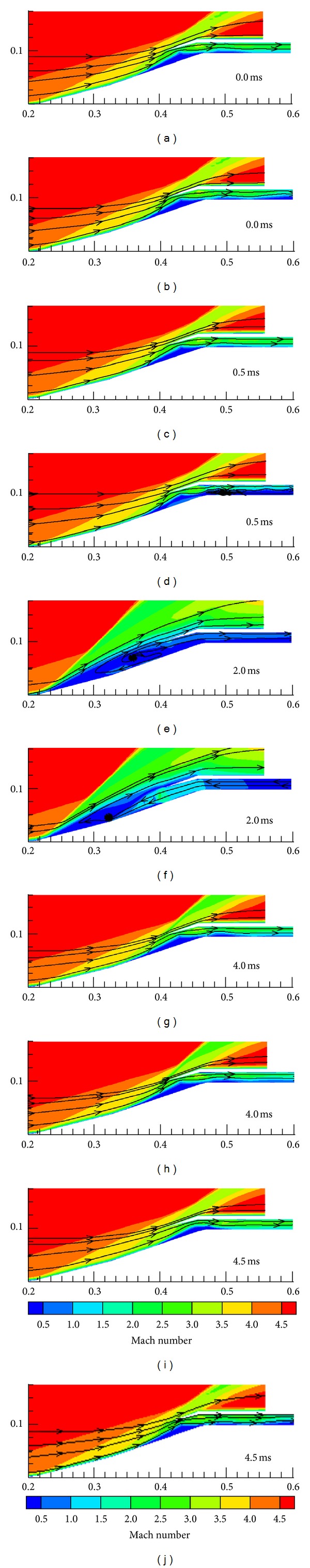
Mach contours mixing with streamlines of cross section *Z*1 (*Z*3) and *Z*2 in a buzz cycle.

Firstly, the unstarted flowfield structure caused by module II throttling was investigated. Because the contour of cross section *Z*1 is the same as that of cross section *Z*3, only one of them is presented. Mach number contours of cross section *Z*1 and *Z*2 in a buzz cycle are shown in Figure 4. 

As can be seen from the figure of *t* = 0 ms in Figure 4, inlet is started though a small separated bubble is formed in entrance. The separated bubble is termed as “initial separated bubble” in order to facilitate the description below. The flowfield structure of the multiple-modules hypersonic inlet goes through four stages as follows.

*Stage I: MassFilling Up (0~0.5 *ms). A high pressure region is formed at the exit of module II due to throttling. To match with the high backpressure, a shock train in the isolator is formed. Then the augment of the backpressure leads to that the shock train moves upstream. At about 0.5 ms, the shock train leading edge is pushed to the inlet throat. The core flow which contains shock train moves downstream while boundary layers shift upstream. The flowfiled structures of module I and module III, however, are distinct with that of module II. And no high backpressure, no shock train, and no reversed flow are formed since there is no throttling at their exits.

*Stage II: Shock System Disgorging (0.5~2.0 *ms). In module II, the shock train is expelled from isolator when backpressure increases to some extents, leading to that the external shock system moves upstream and the captured massflow descends. The gradual increase of adverse pressure gradient results in that the reversed flow occupies more and more part of the internal flowfield until all flow is reversed, and the velocity of reversed flow also increases gradually. In addition, the entrance part of inlet acts as a diverging duct for the reversed flow, and thus, the velocity of the reversed flow increases further. However, no reversed flow emerges in the isolator of module I and module III, while the flow velocity reduces from supersonic to subsonic only a part of airflow enters the isolator due the spillage. It is worth to note that module I and module III are both unstarted and their external shock systems are the same with that of module II, though their internal flowfield structures are distinct.

*Stage III: Shock System Swallowing (2.0~4.0 *ms). Most of airflow spills at stage II. Then due to the imbalance between less captured mass flow and nearly unchanged discharged mass flow, a pressure trough is formed in the exit and propagates upstream, leading to that the shock system moves downstream. At this stage, the reversed flow in the isolator of module II occupies less gradually. However there is no change of flow direction in the isolator of module I and module III, meanwhile flow velocity increases from subsonic to supersonic. Although their internal flowfield structures are distinct, their external shock systems are the same and move downstream together.

*Stage IV: Duct Pressure Recovering (4.0~4.5 *ms). The shock systems reestablish but the pressure of duct is still lower than that at *t* = 0 ms. With the captured airflow increasing, the pressure ascends gradually. At about *t* = 4.5 ms, there is a local separated region at the exit of the isolator, which indicates that the pressure recovers that at *t* = 0 ms. Then the next buzz cycle begins. 

According to the analysis of the four stages, the unstart mechanism can be discussed simply. The occurrence of unstart is caused by the mass flow rate imbalance between inlet entrance and isolator exit. The freestream conditions and the conditions of isolator exit are fixed. The flowfield, however, oscillates all the time. In buzz process, the spillage changes and plays an important role. Thus, the spillage is the major disturbance source for hypersonic inlets, which is also proposed by Tan et al. [13] based on experimental results. 

**Figure 5:**
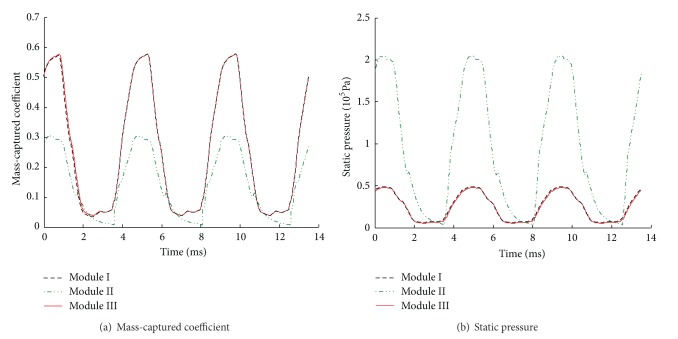
The histories of mass-captured coefficient and static pressure at the exit of isolator.

The histories of mass-captured coefficient and static pressure at the exit of isolator are given in Figure 5. As can be seen from Figure 5, the buzz characteristic of module I is the same with that of module III. The dominant buzz frequencies of three modules are 221.3 Hz, indicating that their unstarted processes are synchronous. The pressure amplitude of module II is higher than that of module I or module III, and the mass flow rate amplitude of module II is lower since the throttling restricts it. Thus unstart phenomenon of module II is much stronger.

**Figure 6:**
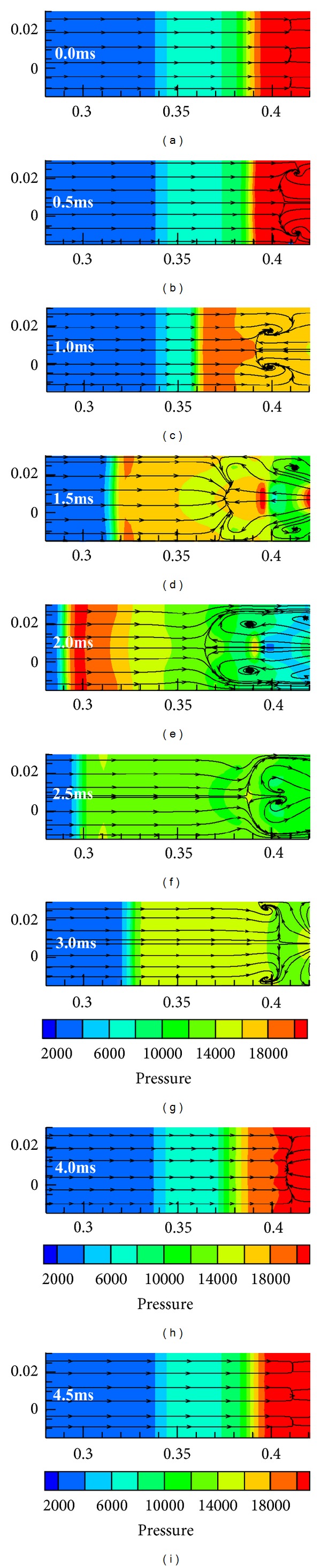
Static pressure contours mixing with streamlines of cross section *Y* in a buzz cycle.

To understand the couple mechanism of unstarted flow for the multiple-modules hypersonic inlet, the flowfield structure of separated bubble in a buzz cycle was analyzed. The pressure contours with streamlines of cross section Y in a buzz cycle are shown in Figure 6. 

It can be noted that reversed streamlines emerge in entrance of all modules as shown in Figure 6(a). The reversed streamlines are located in the initial separated bubble, which have no influence on analysis of coupling mechanism since there is no transverse flow among modules. In cross section Y, the intersection line of streamlines where airflow spills between freestream and reversed flow is termed as spilled line in this paper. Since reversed flow emerges in module II, two weak vortexes are formed, becoming stronger gradually with an increase of reversed flow velocity as shown in Figures 6(b) and 6(c). When the reversed flow velocity is high enough, spilled line is pushed upstream further and extends to both sides, leading to that the two side vortexes are in the downstream of spilled line as shown in Figure 6(d). It is worth to note that a small part flow of vortexes enters side modules. The increase of reversed flow velocity and more airflow of side vortexes entering the side modules due to the upstream movement of external shock system encourage two vortexes to expand further. The middle part of spilled line is pushed upstream while two-sides part is pushed downstream, which leads an arc spilled line to be formed as shown in Figure 6(e). Then external shock system starts moving downstream. The occupation proportion of the reversed flow decreases gradually, and the reversed flow disappears eventually. The end part of vortexes enters all modules and the spilled line is dragged downstream as shown in Figure 6(f). When captured mass flow increases to some extents the initial separated bubble recovers gradually. Thus the reversed flow of initial separated bubble emerges again and its velocity is higher than that of initial state. This reversed flow generates two-side weak vortexes. With the shock system moving downstream further, external shock system is reestablished and the disturbance of initial separated bubble becomes weaker. Thus the two side vortexes is smaller and smaller, until disappear as shown in Figures 6(g) and 6(h). The initial separated bubbles in three modules recover entirely at about 4.5 ms as shown in Figure 6(i). At this time transverse flow disappears, which indicates that there is no effect among three modules. 

### 3.2. Unstarted Flowfield Structure Caused by Module III Throttling

**Figure 7:**
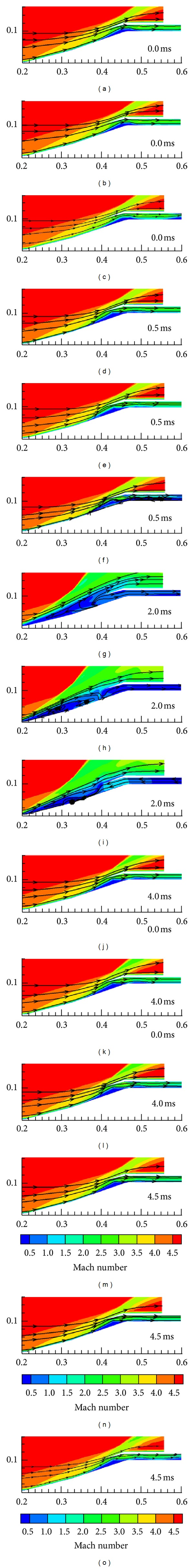
Mach contours mixing with cross section *Z*1, *Z*2, and *Z*3 in a buzz cycle.

Next, the unstarted flowfield structure caused by module III throttling was analyzed.  Figure 7 presents Mach number contours of cross section *Z*1, *Z*2, and *Z*3 in a buzz cycle. It is easy to find that unstarted process includes the same four stages: massfilling up (0~0.5 ms), shock system disgorging (0.5~2.0 ms), shock system swallowing (2.0~4.0 ms), and duct pressure recovering (4.0~4.5 ms).

Similarly, the internal flowfield structures among different modules are distinct. There is shock train formation and reversed flow appearance in the isolator of module III, while these phenomena do not occur in module I and module II. Their external shock systems are identical and are not disturbed by internal flowfield.

**Figure 8:**
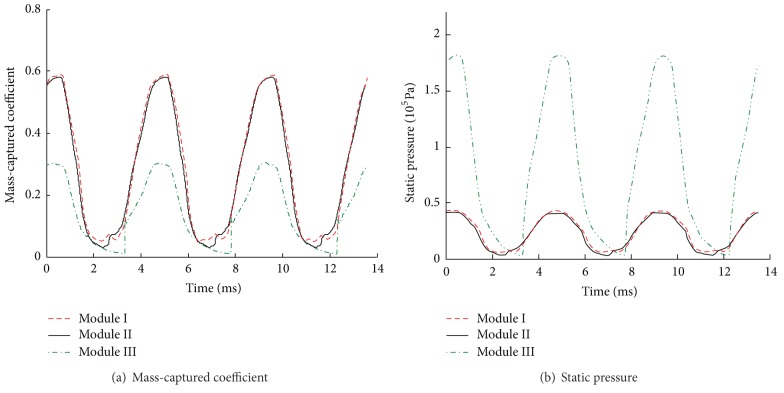
The histories of mass-captured coefficient and static pressure at the exit of isolator.

To understand the buzz differences among three modules further, the histories of mass-captured coefficient and static pressure at the exit are plotted in Figure 8. It is easy to draw a conclusion that buzz processes of three modules are synchronous, and the dominant frequency is 228.9 Hz, while unstart phenomenon of module III is much stronger.

**Figure 9:**
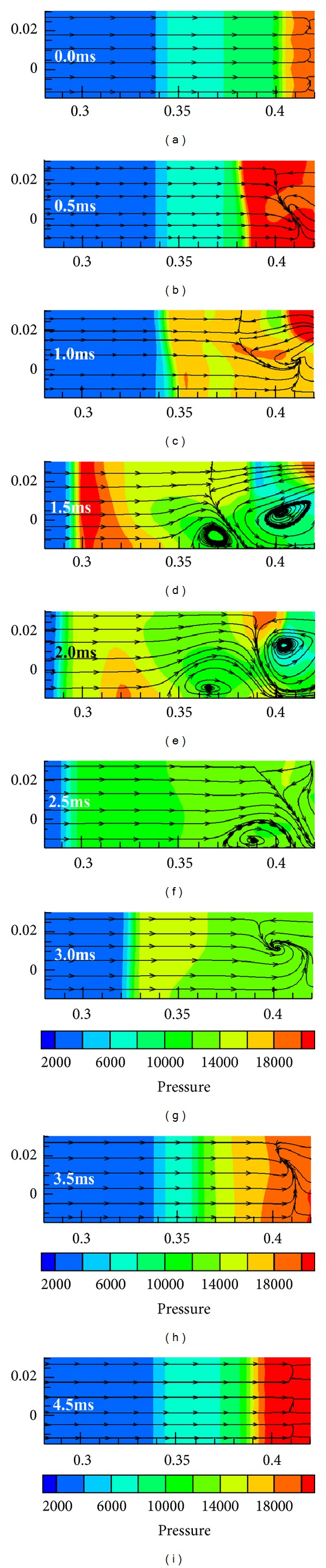
Static pressure contours mixing with streamlines of cross section *Y* in a buzz cycle.

To understand how the unstarted flow of module III results in unstart occurrences in module I and module II, the pressure contours mixing with streamlines of cross section *Y* are analyzed and are presented in Figure 9.

Similarly, three modules do not affect each other since there is no transverse flow among modules at the beginning as shown in Figure 9(a). When the shock train arrives at the throat upstream, reversed flow emerges in module III, leading to that only one weak vortex is formed by the interaction between reversed flow and freestream. With the external shock system moving upstream, the augment of adverse pressure gradient caused by increasing spillage accelerates the reversed flow. Thus the vortex becomes bigger and the spilled line is pushed upstream as shown in Figures 9(b) and 9(c). When the vortex expands to some extent, freestream is chocked, which leads to that a new vortex located upstream of spilled line is formed. Hereafter, the adverse pressure gradient increases slowly since duct pressure is low, resulting in that the velocity of reversed flow increases slowly and more and more airflow enters module I and module II. In addition, freestream is accelerated in the aerodynamics throat which is formed between the two vortexes. Thus, the upstream vortex expands with the external shock system moving upstream further, while the downstream vortex shrinks as shown in Figures 9(d) and 9(e). Then external shock system starts moving downstream due to the formation of pressure trough in module III. The velocity of reversed flow descends with external shock system moving downstream, leading to that the downstream vortex shrinks gradually and disappears eventually as shown in Figure 9(f). At the same time, the chocking effect also decreases gradually due to the shrinkage and the eventual disappearance of downstream vortex, leading the upstream vortex to shrink and vanish. Then a new and weak vortex is formed by the interaction between reversed flow and freestream as shown in Figure 9(g). The initial separated bubble recovers, and reversed flow in the isolator of module III disappears gradually with the external shock system moving downstream further. Thus the transverse flow among three modules is weaker and weaker, and the initial separated bubble recovers entirely at about 4.5 ms as shown in Figure 9(i). At this time, transverse flow disappears, which indicates that there is no interaction among three modules. 

As discussed above, the unstarted coupling mechanism among three modules is concluded below. Vortexes act as a bond among three modules in the whole unstart process. The transverse pressure distribution along *Z*-axis on the cross section *Y* is almost uniform, although a module is chocked. Hence, it has little contribution on the formation of transverse vortexes, and it indicates and verifies that the external shock system is identical at the same time. The reversed flow in isolator, however, dominates the formation, expansion, shrinkage, and disappearance of the vortexes. Thus, it is the major factor of unstart coupling of multiple-modules hypersonic inlet.

## 4. Conclusion

The combination of multiplemodules in parallel manner is an important way to achieve the much higher thrust of scramjet engine, and the unstart coupling mechanism and characteristic among multiple modules hypersonic inlet is an important issue. The unstarted flowfield and coupling characteristic of three-module hypersonic inlet caused by center module II and side module III were conducted, respectively. The results show that the other two modules hypersonic inlet are forced into unstarted flow when unstarted phenomenon appears on a single-module hypersonic inlet due to high backpressure. Vortexes act as a bond among three modules in the whole unstarted process. The transverse pressure distribution on the cross section *Y* caused by throttling is almost uniform, and hence it has little contribution on the formation of transverse vortexes, and it indicates and verifies that the external shock system is identical. However, the reversed flow in isolator dominates the formation, expansion, shrinkage and disappearance of the vortexes, and thus it is the major factor of unstart coupling of multiple modules hypersonic inlet. The coupling effect among multiple modules makes hypersonic inlet be easier to result in unstart.

## Figures and Tables

**Table 1:** Geometric parameters of the hypersonic inlet.

*L*_1_/m	*L*_2_/m	*L*_3_/m	*L*_4_/m	*L*_5_/m	*L*_6_/m
0.212	0.113	0.106	0.169	0.144	0.048

H/m	*δ*_1_/°	*δ*_2_/°	*δ*_3_/°	*δ*_4_/°	*δ*_5_/°

0.015	6	8.3	9.8	13	14.1
